# Therapeutic effect of modified meridian-guided acupoint pressing on lumbar facet joint osteoarthritis: an integrated microbiomics and metabolomics analysis

**DOI:** 10.3389/fmed.2026.1862397

**Published:** 2026-07-08

**Authors:** Yu Jiang, Wanan Qin, Li Wei, Yalian Liao, Gejin Wei

**Affiliations:** 1 Department of Orthopedics, The 923rd Hospital of Joint Logistics Support Force of Chinese People's Liberation Army, Nanning, China; 2 School of Basic Medical Sciences, Guangxi Medical University, Nanning, China

**Keywords:** gut microbiota, inflammation, lumbar facet joint osteoarthritis, metabolomics, modified meridian-guided acupoint pressing, mTORC1 signaling pathway

## Abstract

**Purpose:**

To investigate the therapeutic efficacy of Modified Meridian-Guided Acupoint Pressing (MMGAP) in lumbar facet joint osteoarthritis (LFJ OA) and to explore its underlying mechanisms through integrated microbiomics and metabolomics.

**Methods:**

Animal model study (urokinase-induced LFJ OA in SD rats) with MMGAP intervention, fecal microbiota transplantation (FMT), and mTORC1 inhibitor (rapamycin) validation. Histological, molecular, 16S rRNA sequencing, and UPLC-MS/MS metabolomic analyses were carried out to assess relevant outcomes.

**Results:**

MMGAP significantly reduced inflammatory cell infiltration in lumbar muscles/facet joints, markedly downregulated serum IL-1β and TNF-α (*p* < 0.05) as well as TRPV1 protein expression by >40% at the mRNA and protein levels. It reshaped gut microbiota (significantly elevated Observed Species, Shannon and Chao1 indices, *p* < 0.05; distinct β-diversity clustering vs model group) and serum metabolomic profiles, enriching the mTOR signaling pathway. FMT from MMGAP-treated rats recapitulated therapeutic effects, while rapamycin mimicked MMGAP's anti-inflammatory/analgesic actions.

**Conclusions:**

MMGAP alleviates LFJ OA through gut microbiota reshaping, serum metabolomic reprogramming, and mTORC1 pathway inhibition. These preclinical findings lay preliminary experimental groundwork supporting the research potential of MMGAP as a non-invasive candidate intervention for degenerative joint diseases.

## 1 Introduction

Lumbar facet joint osteoarthritis (LFJ OA) is a prevalent degenerative disorder and a leading cause of chronic low back pain (LBP), affecting over 50% of individuals aged older than 50 years and contributing significantly to global burden of disability (1–3). Current pharmacological interventions, such as non-steroidal anti-inflammatory drugs (NSAIDs) and epidural steroid injections (ESIs), offer only short-term pain relief and are associated with adverse effects, including gastrointestinal complications and cardiovascular adverse events (4–6). Given these limitations, there is a growing interest in exploring alternative therapies, particularly Traditional Chinese Medicine (TCM)-based interventions which has demonstrated efficacy in managing musculoskeletal disorders, including osteoarthritis (OA) (7, 8).

Among TCM modalities, Tuina (Chinese therapeutic massage) has gained recognition for its non-invasive, cost-effective, and patient-friendly properties in musculoskeletal pain management (7, 9). Tuina employs various manipulative techniques—such as pushing, kneading, and rolling—to stimulate acupoints, relax muscles, and improve joint mobility (9). In patients with chronic nonspecific low back pain, Tuina has been shown to significantly reduce pain intensity (as measured by Visual Analog Scale, VAS) and improve functional outcomes (Oswestry Disability Index, ODI) compared to control groups (7). Acupoint stimulation has been demonstrated to exert therapeutic effects on somatic pain (10, 11). However, the therapeutic effects and underlying mechanisms of acupoint massage and pressing in the treatment of LFJ OA remain unclear.

Accumulating studies have verified that gut microbiota participates in the initiation and progression of OA (12, 13). Existing evidence also indicates that acupoint stimulation is capable of modulating gut microbial composition (14). Nevertheless, it remains unclarified whether acupoint pressing improves LFJ OA via remodeling gut microbiota, and its underlying molecular mechanisms are largely unexplored. Accordingly, this study aims to evaluate whether Modified Meridian-Guided Acupoint Pressing (MMGAP) relieves LFJ OA by regulating gut microbiota and further uncover its potential mechanisms through combined 16S rRNA sequencing and metabolomic pathway enrichment analysis.

In the present study, we investigated the therapeutic efficacy and potential mechanisms of MMGAP in lumbar facet joint osteoarthritis (LFJ OA) using rat models, 16S rRNA gene sequencing, and untargeted serum metabolomic profiling. We found that MMGAP significantly alleviated the histopathological lesions and inflammatory responses of LFJ OA, while concurrently reshaping the gut microbiota composition and serum metabolic profiles in LFJ OA rats. Mechanistically, we demonstrated that MMGAP exerted its therapeutic effects via suppression of the mTORC1 signaling pathway. Furthermore, administration of an mTORC1 inhibitor rapamycin significantly ameliorated LFJ OA-related pathological changes and inflammation.

## 2 Materials and methods

### 2.1 Animals

Specific pathogen-free (SPF) male Sprague-Dawley (SD) rats (6 weeks old, 250–300 g) were purchased from SPF Biotechnology Co., Ltd. (Suzhou, China). This study was approved by the Experimental Animal Ethics Committee of Guangxi Medical University (Approval No.: 202507016). The urokinase-induced lumbar facet joint (LFJ) osteoarthritis (OA) model procedure was performed in accordance with previously published reports (15, 16). In brief, rats were anesthetized with isoflurane. After dorsal skin shaving and povidone-iodine disinfection, a 1.5-cm posteromedial longitudinal incision was made along the L3/L4, L4/L5, and L5/L6 intervertebral spaces. The left paravertebral muscles were bluntly dissected to expose the LFJ. A 34-gauge needle connected to a 5-mL microsyringe was used to inject 5 μL of uPA solution (2 mg/mL in normal saline) into each facet joint (L3/L4, L4/L5, L5/L6) of the model and intervention groups; the control group received 5 μL of normal saline per joint. The needle was retained for 15 s to prevent reflux, and the incision was sutured with 4-0 surgical sutures. MMGAP was initiated 24 h after model establishment. Each group contained six rats. Rats were restrained in a custom-made fixator. All MMGAP manipulations were performed with rats placed in a prone position. The pressing sequence is as follows: Baihui (GV20), Sishencong (EX-HN1), Fengchi (GB20), Fengfu (GV16), acupoints of the Conception Vessel (CV), Governor Vessel (GV), and Bladder Meridian (BL) distributed over the erector spinae, followed by distal extremity acupoints including Hegu (LI4) and Bafeng (EX-LE10). Previous studies have demonstrated that stimulation of these acupoints alleviates inflammation and pain (17–22). Pressing was performed with a sterile cotton swab (diameter: 3 mm) at a pressure of 100–110 g (monitored by the MFF multi-point film pressure testing system). Each acupoint was pressed 3 times (5 s per press, 3 s interval). Operators were trained for 1 week to ensure pressure consistency. The intervention was performed once daily for 4 consecutive weeks. For the rapamycin-treated group, rapamycin was intraperitoneally injected daily at 2 mg/kg for four consecutive weeks, while the model group was given an equal volume of vehicle solvent via the same route. All rats were randomly assigned to different groups by random number table after successful LFJ OA modeling. The researchers responsible for histological staining, biochemical detection and data quantification were blinded to group allocation during sample testing and result analysis.

### 2.2 Reagents

Urokinase (uPA, Cat. No.: 9039-53-6; Shanghai Yuanye Biotechnology), Rat IL-1β (JL20884) and TNF-α (JL13202) ELISA kits (Jianglai Biotechnology), All-In-One 5 × RT MasterMix (G592), and Blastaq™ 2 × qPCR MasterMix (G891) were purchased from YEASEN, Jianglai Biotechnology, and abm, respectively. Primary antibodies targeting TRPV1 (A23386; ABclonal) and GAPDH (60004-1-Ig; Proteintech) were used. Rapamycin (CAS No.: 53123-88-9) was purchased from Med Chem Express (MCE).

### 2.3 Fecal Microbiota Transplantation (FMT)

Fresh fecal samples were collected from urokinase-induced LFJ OA rats either treated with or without MMGAP. Samples from each group were pooled to a total of 1 g and suspended in sterile PBS at a concentration of 0.125 g/mL. Recipient rats received a daily intragastric antibiotic cocktail—consisting of vancomycin (50 mg/kg), neomycin sulfate (100 mg/kg), metronidazole (100 mg/kg), and ampicillin (100 mg/kg)—for 5 days prior to transplantation. Recipient rats had established LFJ OA prior to fecal microbiota transplantation. Following the antibiotic regimen, the rats were assigned to one of two groups: the FMT-LFJ OA group, which received fecal suspension from untreated urokinase-induced LFJ OA rats, or the FMT-MMGAP group, which received fecal suspension from MMGAP-treated urokinase-induced LFJ OA rats. Each group was then orally gavaged with 200 μL of the corresponding fecal suspension three times per week.

### 2.4 Histological analysis (H&E staining)

Paraformaldehyde-fixed tissues were dehydrated, cleared, and embedded in paraffin. Serial 5-μm-thick sections were prepared using a microtome, followed by deparaffinization, rehydration, hematoxylin staining (3 min), and eosin staining (30 s). After staining, the sections were re-dehydrated, cleared, and mounted with neutral balsam. Synovitis was semi-quantitatively scored based on three parameters: synovial hyperplasia, subsynovial inflammatory infiltration and angiogenesis. Each parameter was graded from 0 to 3, with the summed total score ranging from 0 to 9. Briefly, synovial hyperplasia was scored according to the number of synovial lining cell layers (0: <3 layers; 1: 3–4 layers; 2: 5–6 layers; 3: >6 layers). Inflammatory infiltration was assessed by lymphocyte distribution (0: no infiltration; 1: scattered lymphocyte aggregation; 2: lymphoid follicle formation; 3: follicles with germinal centers). Angiogenesis was rated as 0 (absent), 1 (mild), 2 (moderate), or 3 (severe), as previously reported (15).

### 2.5 ELISA

Serum IL-1β and TNF-α levels were measured using commercial ELISA kits (Jianglai Biotechnology, Cat. No.: JL20884 for IL-1β; JL13202 for TNF-α) according to the manufacturer's instructions. Briefly, 100 μL of standard or sample was added to pre-coated microplate wells and incubated at 37 °C for 60 min. After washing the wells three times with washing buffer to remove unbound substances, 100 μL of biotinylated detection antibody was added, followed by incubation at 37 °C for 60 min. After a second round of washing (three times), 100 μL of enzyme conjugate was added and incubated at 37 °C for 30 min. Unbound enzyme conjugate was removed by washing three times, and 100 μL of TMB substrate solution was added to each well, followed by incubation at 37 °C for 15 min in the dark. The reaction was terminated by adding 50 μL of stop solution, and the absorbance was measured at 450 nm within 15 min using an ELISA reader (Multiskan FC; Thermo Fisher Scientific). Each sample was assayed in triplicate to ensure reproducibility.

### 2.6 Quantitative PCR (qPCR)

Total RNA was extracted using TRIzol™ reagent, and RNA purity (A260/A280 = 1.8–2.0) was verified. Reverse transcription was performed with All-In-One 5 × RT MasterMix under the following conditions: 37 °C for 15 min, 60 °C for 10 min, and 95 °C for 3 min. Quantitative PCR (qPCR) was conducted with Blastaq™ 2 × qPCR MasterMix on a LightCycler96 system (Roche) using the following thermal cycling protocol: initial denaturation at 95 °C for 3 min, followed by 40 cycles of denaturation at 95 °C for 10 s and annealing/extension at 60 °C for 30 s. Glyceraldehyde-3-phosphate dehydrogenase (GAPDH) was used as the internal reference gene. Relative mRNA expression levels were calculated using the 2^(−ΔΔ*Ct*)^ method. The primer sequences are listed below:

Rat GAPDH forward primer: 5′-CGGCAAGTTCAACGGCACAGTCA-3′

Rat GAPDH reverse primer: 5′-CTTTCCAGAGGGGCCATCCACAG-3′

Rat TRPV1 forward primer: 5′-GAACCCGAGTGCCGACACCTAT-3′

Rat TRPV1 reverse primer: 5′-CACTGCTGCTGTAAGCGATCAC-3′

### 2.7 Western blot

Total protein was extracted with RIPA lysis buffer containing PMSF, and protein concentration was determined by BCA assay. A total of 50 μg of protein per sample lane was denatured, resolved on 12% SDS-PAGE gels, and electrotransferred onto PVDF membranes. Membranes were blocked with 5% non-fat milk (1 h, room temperature), incubated with primary antibodies (GAPDH: 1:5000; TRPV1: 1:1000) at 4 °C overnight, followed by secondary antibodies (1:20,000) for 1 h. Bands were visualized with ECL reagent and quantified using ImageJ software. GAPDH served as the loading control.

### 2.8 Gut microbiota analysis

Fecal microbial DNA was extracted using a FastPure Fecal DNA Isolation Kit (Cat. No.: DC502-01, Vazyme Biotech Co., Ltd., Nanjing, China). The V3-V4 region of the 16S rRNA gene was amplified with primers 343F (5′-TACGGRAGGCAGCAG-3′) and 798R (5′-AGGGTATCTAATCCT-3′). PCR products were purified and sequenced on an Illumina NovaSeq platform. α-diversity (Observed Species, Shannon, Chao1 indices) and β-diversity (NMDS, PCoA) were analyzed using QIIME2 software. LEfSe was used to identify differentially abundant taxa (LDA score > 2.0, *p* < 0.05).

### 2.9 Serum metabolomics analysis

Serum samples (100 μL) were mixed with 400 μL of pre-cooled methanol (−20 °C), vortexed thoroughly for 30 s, and centrifuged at 14,000 × g for 15 min at 4 °C. The supernatants were collected, dried under nitrogen gas, and reconstituted in 100 μL of methanol (chromatographic grade). UPLC-MS/MS analysis was conducted on a Waters ACQUITY UPLC BEH C18 column (2.1 × 50 mm, 1.7 μm) coupled with an ESI-QTRAP mass spectrometer. Orthogonal partial least squares discriminant analysis (OPLS-DA) was employed to distinguish metabolic profiles between groups. Differential metabolites were identified based on the criteria of VIP > 1.0, fold change (FC) > 2.0 or <0.5, and *p* < 0.05. Kyoto Encyclopedia of Genes and Genomes (KEGG) pathway enrichment analysis was subsequently performed to explore the functional relevance of these differential metabolites.

### 2.10 Statistical analysis

Data were analyzed using SPSS 26.0 and GraphPad Prism 8.0.1 and are expressed as mean ± standard error of the mean (SEM). Normality and homogeneity of variance were assessed via the Shapiro-Wilk test and Levene's test, respectively. Intergroup comparisons between two groups were conducted with the unpaired Student's *t*-test; comparisons across multiple groups were analyzed using one-way ANOVA followed by Sídák's post-hoc test. A *p* value <0.05 was defined as statistically significant.

## 3 Results

### 3.1 Modified meridian-guided acupoint pressing alleviates urokinase-induced lumbar facet joint osteoarthritis

**Figure 1:**
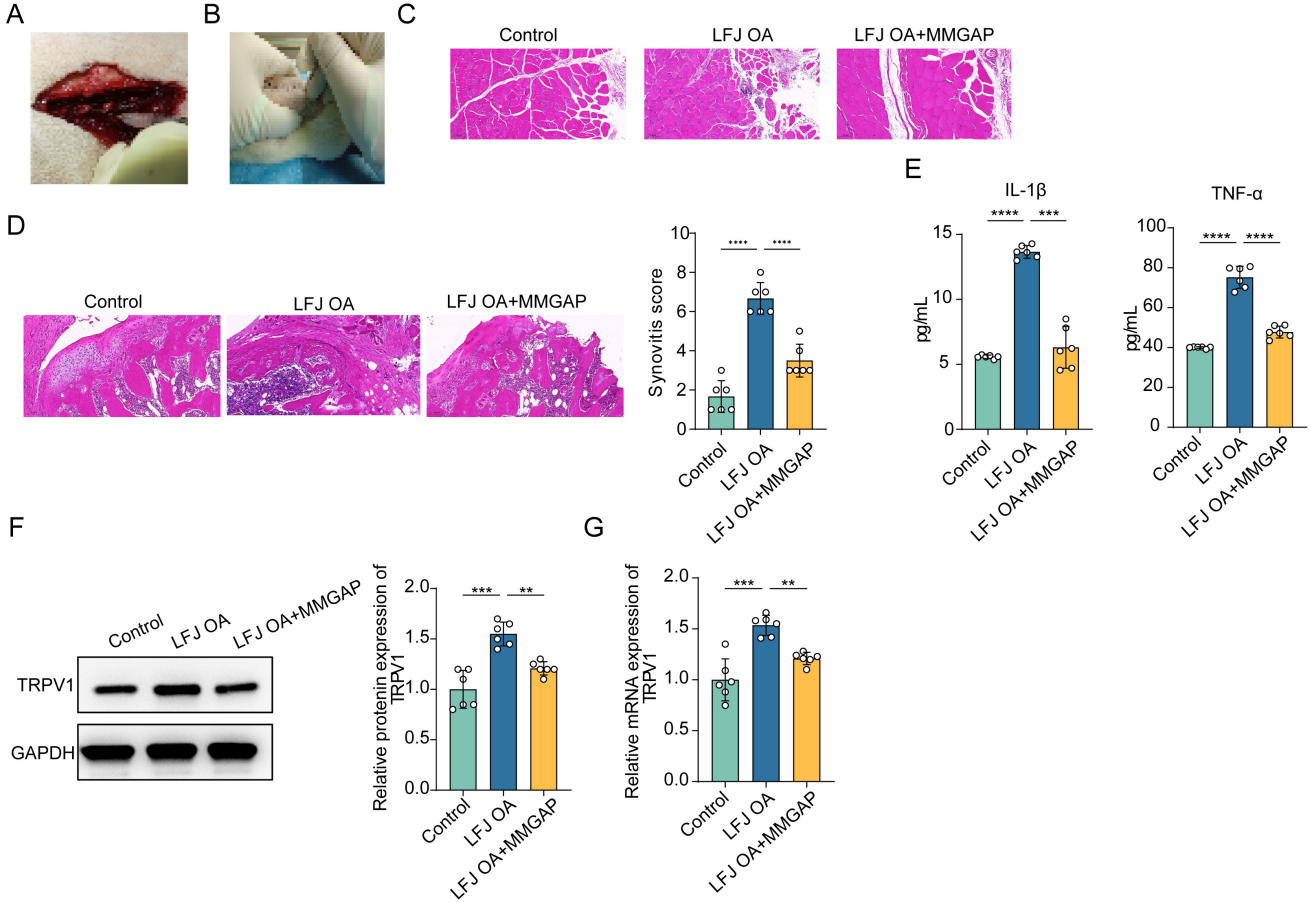
MMGAP alleviates urokinase-induced lumbar facet joint osteoarthritis. **(A)** Schematic diagram of intra-articular injection of urokinase into the lumbar facet joints. **(B)** Schematic diagram of Modified Meridian-guided Acupoint Pressing therapy. **(C)** Representative H&E-stained histological images of the erector spinae muscles. **(D)** Representative H&E-stained histological images of lumbar facet joints. Semi-quantitative scoring was performed to evaluate the degree of synovitis. **(E)** Serum levels of IL-1β and TNF-α in rats from each group were measured by ELISA. **(F)** The expression of TRPV1 in the erector spinae muscle tissue was detected by Western blot. **(G)** The mRNA expression of TRPV1 in the erector spinae muscle tissue was detected by qPCR. Statistical analysis for **(E**, **F)**, and **(G)** was performed using one-way analysis of variance (ANOVA) followed by Sidak's correction for multiple comparisons. All data are presented as the mean ± standard error of the mean (SEM). ****p < 0.0001, ***p < 0.001, and **p < 0.01.

LFJ OA was induced via intra-articular urokinase injection into the lumbar facet joints (Figure 1A). Rats in the treatment group received modified meridian-guided acupoint pressing (MMGAP) intervention (Figure 1B). Following 4 weeks of intervention, inflammatory changes within lumbar muscles and facet joints were evaluated. H&E staining showed prominent inflammatory cell infiltration in the lumbar muscles of the model group; by contrast, MMGAP markedly diminished inflammatory cell accumulation at the muscle-fascia interface (Figure 1C). Likewise, facet joints from model rats exhibited robust inflammatory infiltration, an abnormality effectively suppressed by MMGAP administration (Figure 1D). Serum proinflammatory cytokine quantification confirmed that MMGAP significantly reduced circulating interleukin-1β (IL-1β) and tumor necrosis factor-α (TNF-α) concentrations in urokinase-induced LFJ OA rats (Figure 1E). Transient receptor potential vanilloid 1 (TRPV1), a cation channel linked to nociception and inflammation, is known to mediate osteoarthritic pain signaling. We therefore further examined MMGAP's regulatory effect on TRPV1 expression. Western blot results indicated elevated TRPV1 abundance in lumbar muscle of model animals, whereas MMGAP treatment substantially downregulated its expression (Figures 1F, G). Collectively, these preclinical observations suggest that MMGAP relieves local inflammatory responses and blocks pain-transducing signaling in urokinase-triggered LFJ OA.

### 3.2 Effects of MMGAP on gut microbiota in LFJ OA

**Figure 2:**
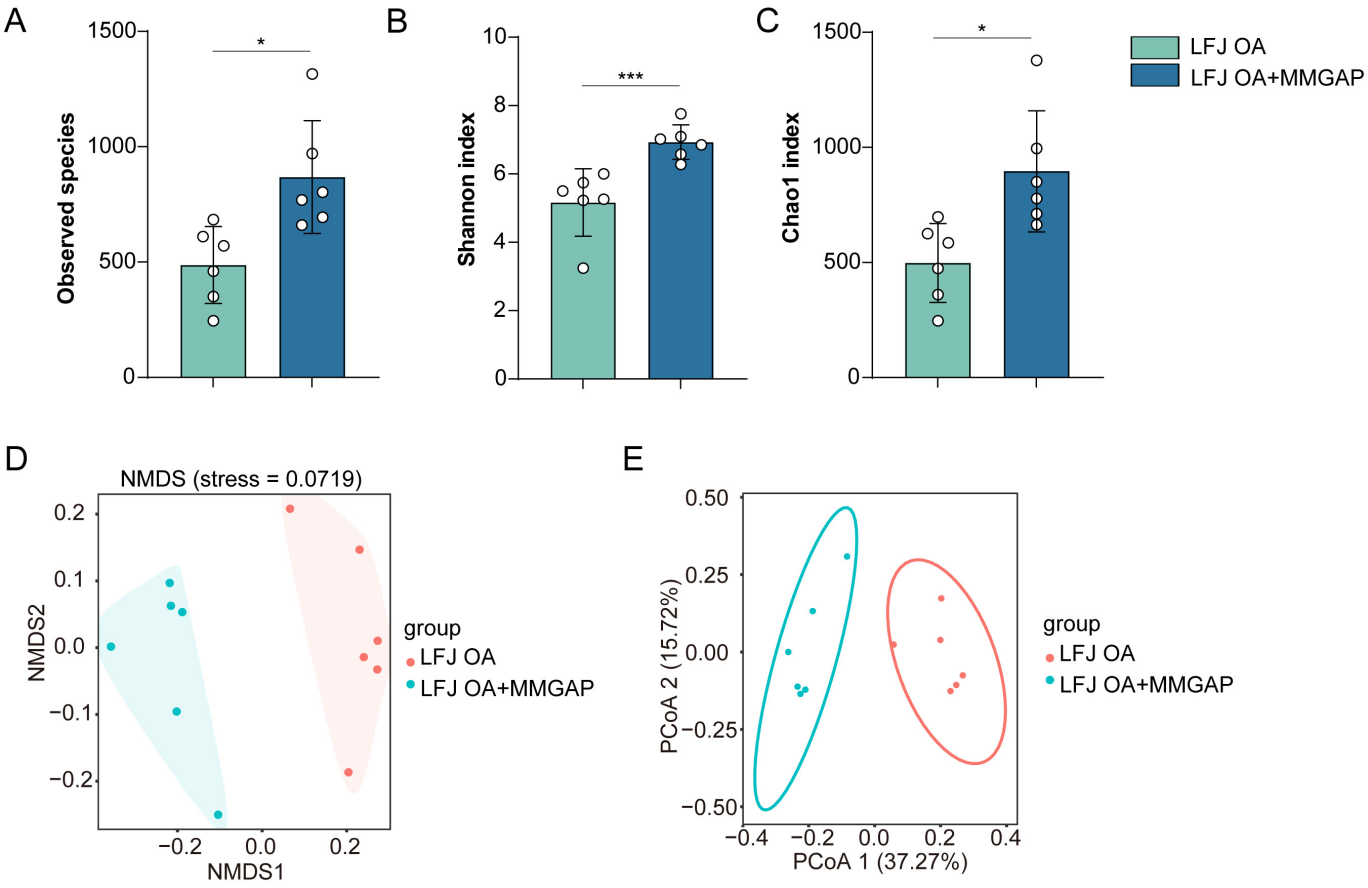
Effects of MMGAP on gut microbiota in LFJ OA. **(A–C)** Alpha diversity of the gut microbiota was analyzed using the Observed species **(A)**, Shannon **(B)**, and Chao1 **(C)** indexes; statistical comparisons were performed by unpaired Student's *t*-test. **(D)** NMDS analysis was performed based on Bray-Curtis dissimilarity to visualize differences in gut microbial community structure between the LFJ OA group and the LFJ OA + MMGAP group. **(E)** PCoA was conducted to compare gut microbial community composition between the LFJ OA group and the LFJ OA + MMGAP group. All data are presented as the mean 1 standard error of the mean (SEM). ***p < 0.001 and *p < 0.05.

**Figure 3:**
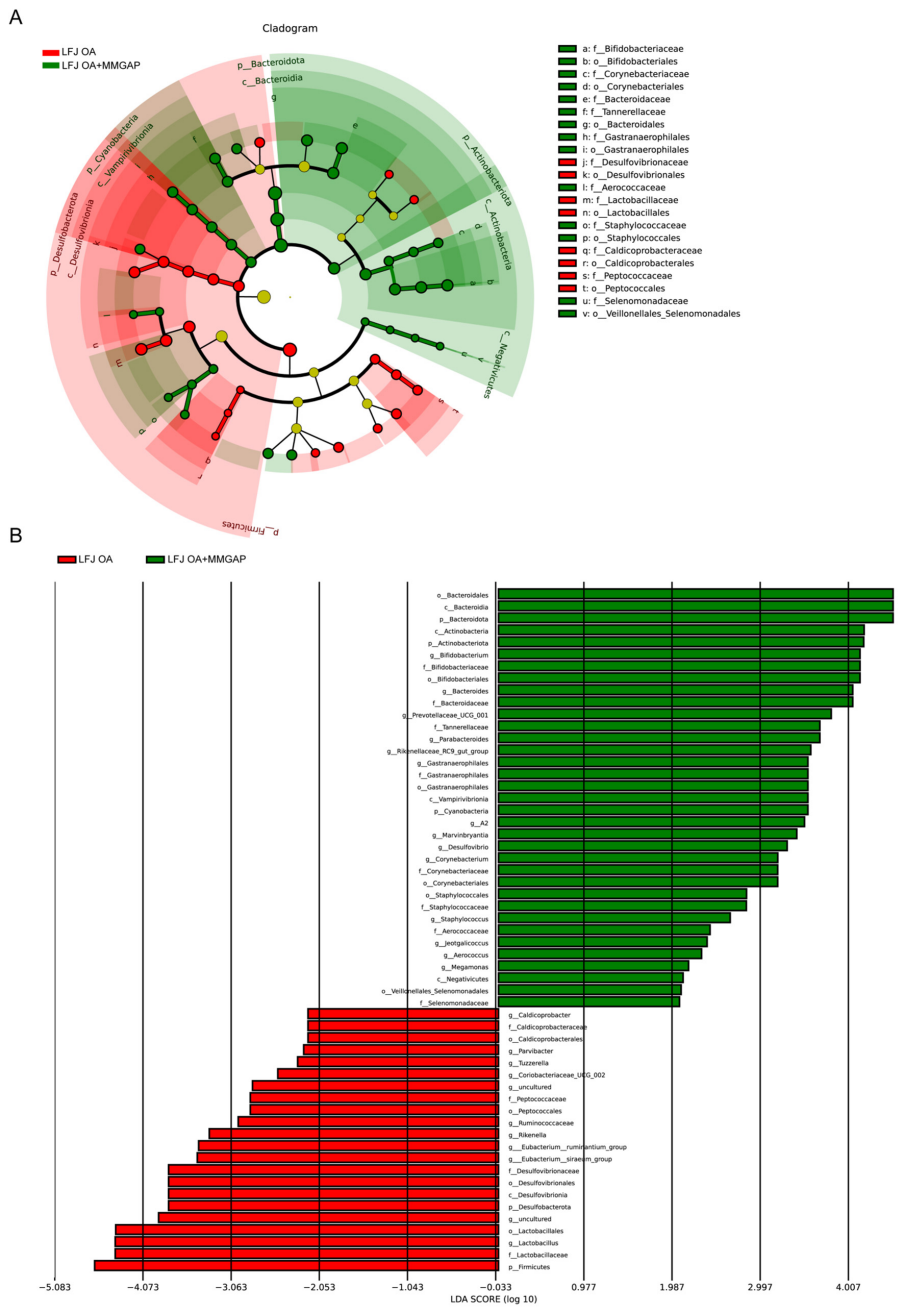
LEfSe **(A)** and phylogenetic tree-based **(B)** analysis were conducted to identify differentially abundant bacterial taxa among the groups.

Previous studies have demonstrated that TCM physical therapies, such as tuina (Chinese massage) and acupuncture, can modulate the gut microbiota—a key contributor to the pathogenesis of LFJ OA. We therefore further explored MMGAP's regulatory effect on the gut microbiota in LFJ OA rats. Our results showed that MMGAP enhanced gut microbial α-diversity in LFJ OA rats, as evidenced by significant increases in the Observed Species, Shannon, and Chao1 indices (Figures 2A–C). Subsequent β-diversity analysis revealed that non-metric multidimensional scaling (NMDS) and principal coordinate analysis (PCoA) exhibited distinct clustering of microbial community structures between MMGAP-treated rats and LFJ OA model rats (Figures 2D, E), indicating a marked shift in gut microbiota composition. Additionally, linear discriminant analysis effect size (LEfSe) and phylogenetic investigation of communities by reconstruction of unobserved states (PICRUSt) were employed to identify bacterial taxa with differential abundances among groups (Figures 3A, B). The results indicated that multiple bacterial taxa displayed altered relative abundances in the MMGAP group compared with the LFJ OA group. Collectively, these preclinical findings suggest that MMGAP treatment can significantly remodel the gut microbiota composition in urokinase-induced LFJ OA rats.

### 3.3 FMT from MMGAP-treated rats ameliorates LFJ OA

**Figure 4:**
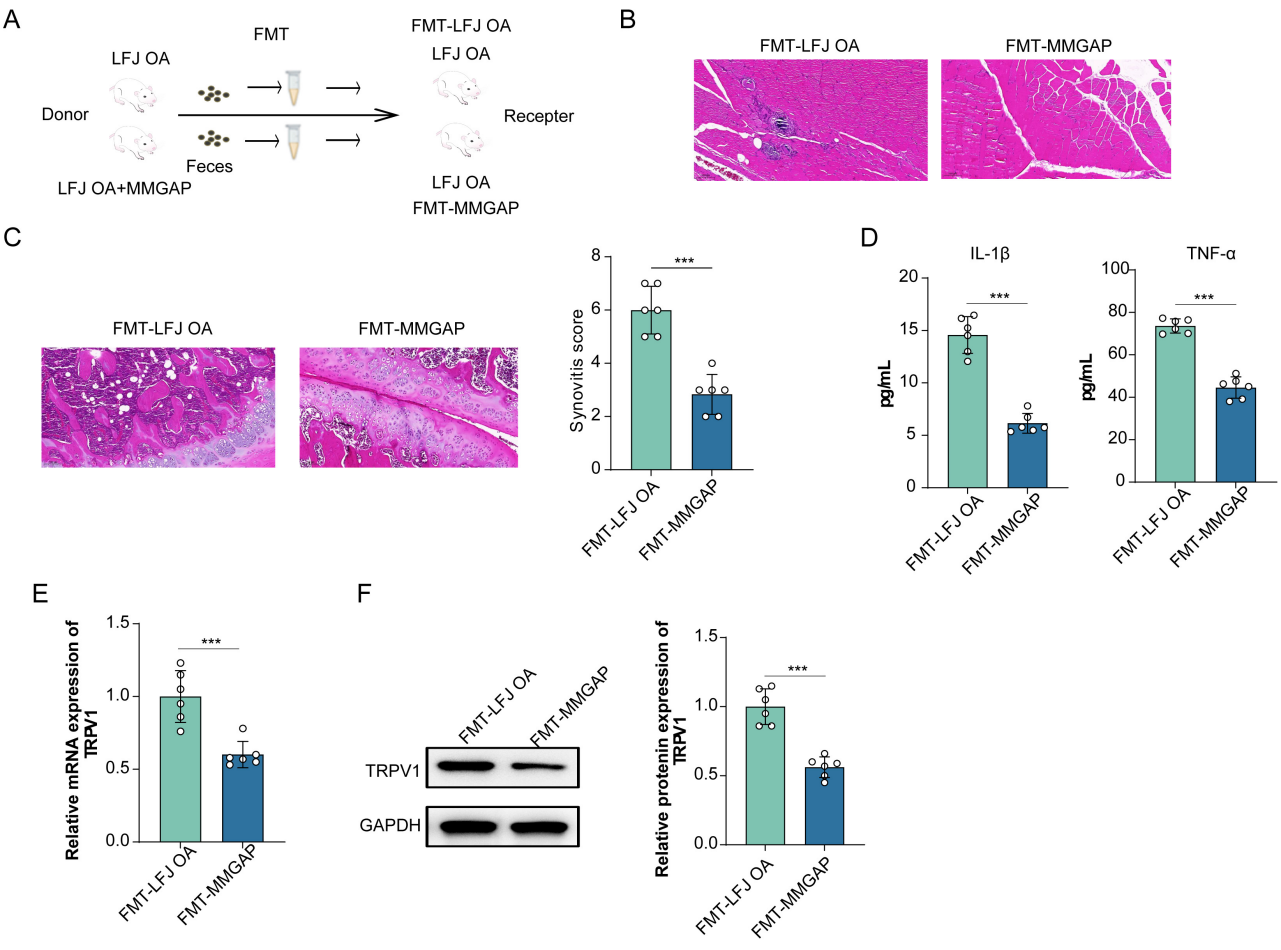
Effect of fecal microbiota transplantation (FMT) from LFJ OA + MMGPA group on LFJ OA. (A) Graphical abstract of the FMT study. (B) Representative H&E-stained histological images of the erector spinae muscles. (C) Representative H&E-stained histological images of lumbar facet joints. Semi-quantitative scoring was performed to evaluate the degree of synovitis. (D) Serum levels of IL-1β and TNF-α in rats from each group were measured by ELISA. (E) The mRNA expression of TRPV1 in the erector spinae muscle tissue was detected by qPCR. (F) The expression of TRPV1 in the erector spinae muscle tissue was detected by Western blot. Statistical analysis for (D, E), and (F) was performed using unpaired Student's t-test. All data are presented as the mean ± standard error of the mean (SEM). ***p <0.001.

FMT has been previously confirmed to slow osteoarthritis progression. Having verified that MMGAP remodels the gut microbiota in LFJ OA rats, we further explored the therapeutic efficacy of FMT against LFJ OA. Specifically, fecal microbiota harvested from MMGAP-treated donor rats was transplanted into LFJ OA recipient rats (Figure 4A). The results revealed that FMT derived from MMGAP-treated donors markedly attenuated inflammatory infiltration in the lumbar muscles and lumbar facet joints of LFJ OA rats (Figures 4B, C). Meanwhile, this intervention significantly lowered serum levels of the pro-inflammatory cytokines IL-1β and TNF-α (Figure 4D). Moreover, FMT from MMGAP-exposed animals suppressed TRPV1 protein expression in the lumbar muscle of recipient rats (Figures 4E, F). Collectively, these preclinical findings indicate that fecal microbiota transplantation from MMGAP-treated donors ameliorates urokinase-induced LFJ OA in recipient animals.

### 3.4 MMGAP modulates serum metabolites in LFJ OA rats

**Figure 5:**
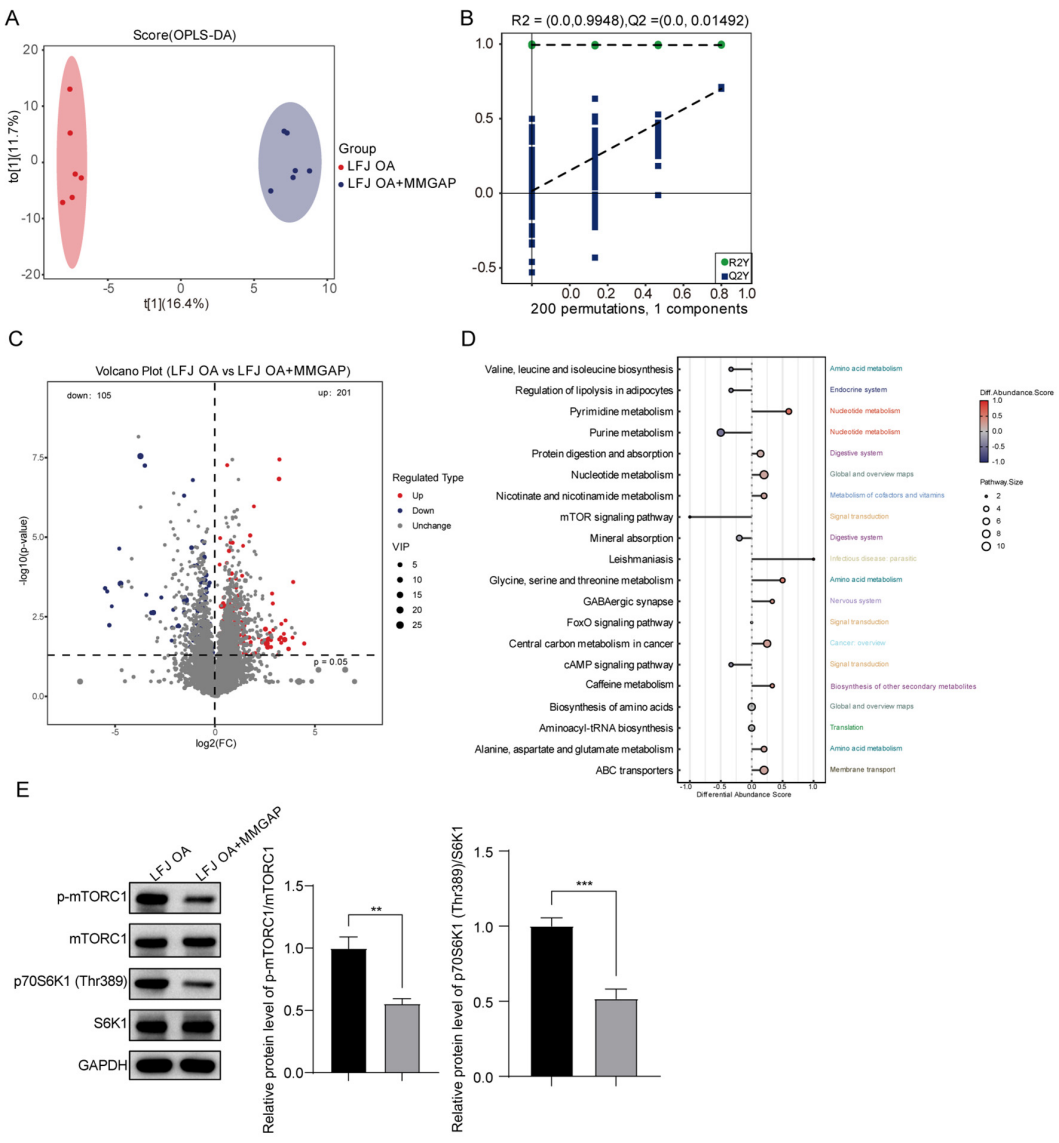
Serum metabolomics analysis of the LFJ OA and LFJ OA + MMGPA groups. **(A**, **B)** OPLS-DA score plot **(A)** and permutation test **(B)** for the comparison between the LFJ OA and LFJ OA + MMGPA groups. **(C)** Volcano plot comparing the LFJ OA and LFJ OA + MMGPA groups; criteria for identifying significant differences were defined as VIP > 1.0, FC > 2 or <0.5, and *p* < 0.05. **(D)** KEGG pathway enrichment differential abundance score plot. **(E)** The phosphorylation levels of mTORC1 as well as its downstream effector p70S6K1 (Thr389) within the lumbar tissues of LFJ OA rats were detected by Western blot. Statistical analysis for **(E)** was performed using unpaired Student's *t*-test. All data are presented as the mean ± standard error of the mean (SEM). ***p < 0.001 and **p < 0.01.

To further explore how MMGAP modulates serum metabolites in LFJ OA rats, we conducted untargeted metabolomic profiling of serum specimens. Metabolomic datasets were processed via orthogonal partial least squares–discriminant analysis (OPLS-DA). Score plots showed evident separation across the control, LFJ OA model, and MMGAP-treated cohorts, verifying divergent group-specific metabolic signatures (Figure 5A). Permutation testing confirmed OPLS-DA model validity; the resulting R^2^ and Q^2^ values followed standard trends, supporting robust model stability and predictive capacity (Figure 5B). Volcano plots were used to screen differential metabolites based on predefined thresholds: VIP > 1.0, FC > 2.0 or <0.5, and *p* < 0.05. Relative to the LFJ OA model group, 201 metabolites were significantly upregulated whereas 105 were downregulated in the MMGAP group (Figure 5C). The top 20 significantly increased and top 20 decreased differential metabolites are summarized in Supplementary Tables 1 and 2. KEGG pathway enrichment analysis, illustrated with a DA score plot, identified the mTOR signaling pathway as the most prominently enriched pathway linked to altered metabolism (Figure 5D). Furthermore, our data demonstrate that MMGAP decreases the phosphorylation levels of mTORC1 as well as its downstream effector p70S6K1 (Thr389) within the lumbar tissues of LFJ OA rats (Figure 5E). Collectively, these preclinical results indicate that MMGAP treatment substantially remodels the serum metabolome of LFJ OA rats, with the mTOR cascade serving as a core regulatory target.

### 3.5 mTOR inhibitor rapamycin attenuates urokinase-induced LFJ OA

**Figure 6:**
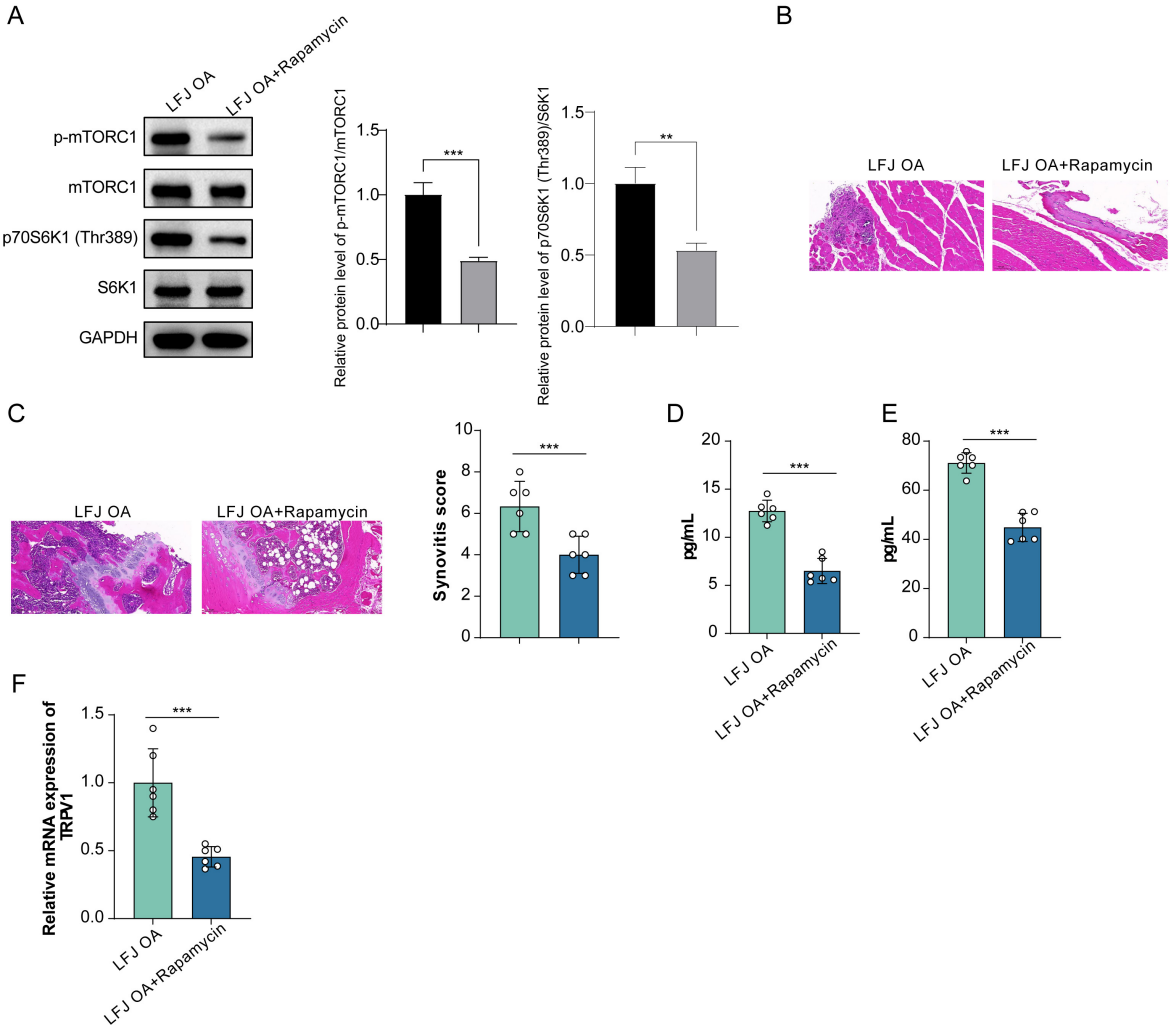
Rapamycin Attenuates LFJ OA. **(A)** The phosphorylation levels of mTORC1 as well as its downstream effector p70S6K1 (Thr389) within the lumbar tissues of LFJ OA rats were detected by Western blot. **(B)** Representative H&E-stained histological images of the erector spinae muscles. **(C)** Representative H&E-stained histological images of lumbar facet joints. Semi-quantitative scoring was performed to evaluate the degree of synovitis. **(D, E)** Serum levels of IL-1β and TNF-α in rats from each group were measured by ELISA. **(F)** The mRNA expression of TRPV1 in the erector spinae muscle tissue was detected by qPCR. Statistical analysis for **(A**, **D–F)** was performed using unpaired Student's *t*-test. All data are presented as the mean ± standard error of the mean (SEM). ***p < 0.001 and **p < 0.01.

Given that MMGAP was confirmed to inhibit the mTOR signaling pathway, we further evaluated the therapeutic efficacy of rapamycin, a specific inhibitor of the mTORC1 pathway, in LFJ OA rats. The results showed that rapamycin treatment significantly attenuated phosphorylation levels of mTORC1 and its downstream p70S6K1 (Thr389) in lumbar tissues of LFJ OA rats (Figure 6A). Further, rapamycin treatment significantly attenuated inflammatory infiltration in the lumbar muscles and facet joints of LFJ OA rats, which was consistent with the anti-inflammatory effect of MMGAP (Figures 6B, C). Concomitantly, rapamycin administration remarkably reduced serum levels of the pro-inflammatory cytokines IL-1β and TNF-α compared with the LFJ OA model group (Figures 6D, E). Furthermore, rapamycin significantly downregulated TRPV1 expression in the lumbar muscle tissues of LFJ OA rats (Figure 6F). Collectively, these preclinical findings indicate that inhibition of the mTOR signaling pathway effectively mitigates inflammatory responses and suppresses pain-transducing signals in LFJ OA rats.

## 4 Discussion

LFJ OA remains a major contributor to chronic low back pain and disability globally, as current pharmacological interventions provide only limited long-term benefits owing to their inherent adverse effects. TCM physical therapies, such as Tuina (Chinese therapeutic massage) and acupoint stimulation, have emerged as promising non-invasive alternatives for the management of musculoskeletal disorders (23, 24), but their therapeutic mechanisms in LFJ OA have not been fully elucidated. The present study, integrating microbiomic, metabolomic, and molecular biological approaches, provides preclinical evidence that MMGAP alleviates LFJ OA via gut microbiota remodeling, serum metabolic reprogramming, and suppression of the mTORC1 signaling pathway.

Conventional Tuina involves various kneading, rolling and rubbing manipulations with unfixed pressure(25), whereas the MMGAP applied in this study uses fixed-point constant compression alone without extra auxiliary massage movements. Our results showed that MMGAP significantly reduced inflammatory cell infiltration in the lumbar muscles and facet joints, downregulated the pro-inflammatory cytokines IL-1β and TNF-α, and suppressed TRPV1 protein expression. These findings are consistent with previous studies demonstrating the anti-inflammatory and analgesic effects of acupoint stimulation in inflammatory musculoskeletal pain (26–28). TRPV1, a key mediator of pain transmission in osteoarthritis (27), is known to be upregulated in inflamed tissues and contributes to nociceptive signaling (29); the downregulation of TRPV1 by MMGAP highlights its potential to target pain pathways at the molecular level. Notably, both MMGAP and conventional Tuina exert therapeutic effects against chronic low back pain; however, MMGAP's meridian-guided acupoint selection (e.g., GV20, GB20, and BL meridians) relies on a more targeted theoretical framework rooted in Traditional Chinese Medicine, which differentiates it from generalized non-specific massage. This interpretation complies with the TCM principle of “meridian regulation”, which holds that stimulation of designated acupoints regulates qi and blood circulation to ameliorate musculoskeletal disorders (30). Thus, MMGAP represents a more precise acupoint pressing approach for the treatment of chronic nonspecific low back pain.

The gut–joint axis has emerged as a critical regulatory axis in OA pathogenesis, wherein gut microbiota dysbiosis drives systemic inflammation and subsequent articular degeneration (31, 32). Our study demonstrated that MMGAP significantly reshaped the gut microbiota composition in LFJ OA rats, as evidenced by altered α-diversity (Observed Species, Shannon, Chao1 indices) and distinct β-diversity clustering. Furthermore, FMT from MMGAP-treated rats recapitulated the therapeutic effects, including reduced inflammation and TRPV1 expression, suggesting that gut microbiota modulation plays an important role in the therapeutic action of MMGAP. This is consistent with recent findings that TCM external therapies, such as Tuina (33, 34). The differentially abundant taxa identified by LEfSe may include potential probiotics or pathobionts; future studies should characterize their functional roles. However, a methodological limitation should be noted that an “antibiotics + sterile PBS” control group was not included. Antibiotic exposure alone may alter gut barrier function, systemic immune status, and local inflammatory responses (35), which could potentially confound the observed therapeutic outcomes. Future studies should include relevant control groups to rule out the confounding effects of antibiotics. Although we characterized the shifts in gut microbial composition induced by MMGAP, the current study failed to verify the exact biological function of distinct differential bacterial taxa. Future studies are warranted to explore the specific roles of these pivotal bacterial taxa in the pathogenesis and progression of LFJ OA. Collectively, our results demonstrate that MMGAP remodels the gut microbiota composition in LFJ OA rats, and fecal microbiota harvested from MMGAP-treated animals can alleviate inflammatory responses in recipient LFJ OA rats.

Untargeted metabolomics revealed that MMGAP altered 306 serum metabolites and enriched the mTOR signaling pathway, a key regulator of inflammation, autophagy, and cell metabolism in OA (36, 37). Previous studies have shown that inhibiting mTOR and thereby promoting autophagy in chondrocytes alleviates osteoarthritis (38); mTOR inhibition with rapamycin prevents senescence and effectively treat osteoarthritis (39). Our results in rat models confirmed that rapamycin partially reproduced the anti-inflammatory effect and downregulation of TRPV1, suggesting that suppression of the mTORC1 pathway serves as one critical mechanistic contributor underlying MMGAP-mediated protection against LFJ OA. Importantly, the gut microbiota-metabolome axis may link MMGAP to mTOR regulation: gut microbiota-derived metabolites (e.g., SCFAs, bile acids) are known to modulate mTOR signaling in host tissues (40, 41), suggesting that MMGAP-induced microbiota changes may alter metabolite production to inhibit mTORC1. Future work will further explore how serum metabolites modulate the mTOR cascade to regulate the progression of LFJ OA.

Given the promising preclinical anti-inflammatory and microbiota-modulating effects of MMGAP observed in LFJ OA rats, exploring its potential clinical translation is of considerable significance. For human application, MMGAP could be standardized as follows: First, the treatment procedure should be adjusted based on human anatomical characteristics—acupoints including Baihui (GV20), Sishencong (EX-HN1), Fengchi (GB20), and Fengfu (GV16) would remain core targets, while CV/GV/BL acupoints in the erector spinae region should be localized according to individual body proportions, with Hegu (LI4) and Bafeng (EX-LE10) retained as key extremity points. The manipulation should adopt a standardized pressing intensity (graded as mild-to-moderate, defined as the pressure evoking a slight local skin hyperemia without pain) and rhythmic technique (circular pressing with 2–3 cycles per second). Second, the recommended treatment duration could reference preclinical intervention cycles: a 4–6-week course with 3 sessions per week (each session lasting 15–20 min) is proposed. Third, practical considerations for healthcare professionals include: (1) individualized acupoint adjustment for patients with anatomical variations or comorbidities (e.g., avoiding excessive pressure on CV acupoints in pregnant individuals); (2) training in standardized pressure control to ensure consistency across practitioners; (3) combination with clinical assessment tools (e.g., VAS for pain, WOMAC for OA severity) to monitor treatment response and optimize protocols dynamically. It should be emphasized that these proposals are preliminary and require validation in future clinical trials to confirm safety, efficacy, and optimal standardization parameters for human LFJ OA patients.

## 5 Limitations

The intra-articular uPA-induced OA model can reliably recapitulate key OA pathological features including progressive cartilage degeneration, synovial hyperplasia and inflammation, with simple operation and good reproducibility (15, 16). However, it mainly depends on uPA-mediated cartilage matrix degradation and cannot fully mimic the multifactorial etiology of spontaneous age-related OA. Moreover, the present study did not perform toluidine blue staining and micro-CT detection, leading to insufficient comprehensive evaluation of cartilage and subchondral bone lesions. Future studies will further evaluate more comprehensive indicators of this model by supplementing toluidine blue staining to assess cartilage proteoglycan loss and micro-CT analysis to observe subchondral bone remodeling and osteophyte formation. In addition, microbiota composition was not detected after FMT in the present study, and relevant microbiota profiling should be performed following FMT in future investigations. Besides, behavioral pain assessments were absent in the current study, and related behavioral pain evaluation will be included in subsequent experimental designs.

## 6 Conclusion

In conclusion, our study demonstrates that MMGAP alleviates LFJ OA through a novel mechanism involving gut microbiota reshaping, serum metabolomic reprogramming, and mTORC1 pathway inhibition. These preclinical findings based on rat experiments offer preliminary experimental evidence supporting the therapeutic prospect of MMGAP against LFJ OA in animal models; further well-designed clinical studies are still required to verify its clinical efficacy and practical application in human patients with LFJ OA.

## Data Availability

The 16S rRNA sequencing data have been deposited in the NCBI database under BioProject accession number PRJNA1480653 (https://www.ncbi.nlm.nih.gov/bioproject/PRJNA1480653). The untargeted metabolomics data have been deposited in the MetaboLights repository under accession number MTBLS14816 (https://www.ebi.ac.uk/metabolights/reviewer6eaa7bab-3635-48db-a12b-11eebb8c1724).
