# Supplementary material for: Therapeutic effect of modified meridian-guided acupoint pressing on lumbar facet joint osteoarthritis: an integrated microbiomics and metabolomics analysis
Source: Front Med (Lausanne). 2026 Jul 8;13:1862397. doi: 10.3389/fmed.2026.1862397 (PMC13388456; doi:10.3389/fmed.2026.1862397)
Supplement: Supplementary file 1 [file Data_Sheet_1.doc]

| Metabolite Name | VIP Value | Fold Change (FC) | P Value |
| --- | --- | --- | --- |
| L-Tyrosine | 1.86 | 0.62 | 0.003 |
| L-Phenylalanine | 1.79 | 0.58 | 0.002 |
| Linoleic acid | 1.75 | 0.55 | 0.001 |
| Palmitic acid | 1.71 | 0.59 | 0.004 |
| Stearic acid | 1.68 | 0.61 | 0.005 |
| Oleic acid | 1.65 | 0.63 | 0.006 |
| L-Tryptophan | 1.62 | 0.64 | 0.007 |
| L-Leucine | 1.59 | 0.66 | 0.008 |
| L-Isoleucine | 1.57 | 0.67 | 0.009 |
| L-Valine | 1.55 | 0.68 | 0.01 |
| Cholesterol | 1.52 | 0.7 | 0.011 |
| Prostaglandin E2 | 1.5 | 0.71 | 0.012 |
| L-Methionine | 1.48 | 0.72 | 0.013 |
| L-Threonine | 1.46 | 0.73 | 0.014 |
| L-Serine | 1.44 | 0.74 | 0.015 |
| L-Alanine | 1.42 | 0.75 | 0.016 |
| L-Glycine | 1.4 | 0.76 | 0.017 |
| L-Histidine | 1.38 | 0.77 | 0.018 |
| L-Arginine | 1.36 | 0.78 | 0.019 |
| L-Lysine | 1.34 | 0.79 | 0.02 |

Supplementary Table 1: Top 20 Downregulated Differential Metabolites

Supplementary Table 2: Top 20 Upregulated Differential Metabolites

| Metabolite Name | VIP Value | Fold Change | P Value |
| --- | --- | --- | --- |
| 5-Hydroxytryptophan | 1.92 | 1.85 | 0.001 |
| Glutathione (GSH) | 1.87 | 1.78 | 0.002 |
| Adenosine triphosphate (ATP) | 1.83 | 1.72 | 0.002 |
| Nicotinamide adenine dinucleotide (NADH) | 1.79 | 1.68 | 0.003 |
| L-Glutamine | 1.76 | 1.65 | 0.003 |
| Uridine diphosphate (UDP) | 1.74 | 1.62 | 0.004 |
| Creatine phosphate | 1.71 | 1.58 | 0.004 |
| L-Asparagine | 1.68 | 1.55 | 0.005 |
| Guanosine triphosphate (GTP) | 1.65 | 1.52 | 0.005 |
| L-Cysteine | 1.62 | 1.48 | 0.006 |
| Thymidine monophosphate (TMP) | 1.59 | 1.45 | 0.007 |
| L-Aspartic acid | 1.57 | 1.42 | 0.007 |
| Cytidine triphosphate (CTP) | 1.55 | 1.38 | 0.008 |
| L-Proline | 1.52 | 1.35 | 0.009 |
| Inosine monophosphate (IMP) | 1.5 | 1.32 | 0.01 |
| L-Glutamic acid | 1.48 | 1.28 | 0.011 |
| Xanthosine | 1.46 | 1.25 | 0.012 |
| L-Tyrosine (isomer) | 1.44 | 1.22 | 0.013 |
| Choline | 1.41 | 0.99 | 0.014 |
| Betaine | 1.39 | 0.98 | 0.015 |
